# Short-term outpatient follow-up of COVID-19 patients: A multidisciplinary approach

**DOI:** 10.1016/j.eclinm.2021.100731

**Published:** 2021-01-28

**Authors:** M.A. de Graaf, M.L. Antoni, M.M. ter Kuile, M.S. Arbous, A.J.F. Duinisveld, M.C.W. Feltkamp, G.H. Groeneveld, S.C.H. Hinnen, V.R. Janssen, W.M. Lijfering, S. Omara, P.E. Postmus, S.R.S. Ramai, N. Rius-Ottenheim, M.J. Schalij, S.K. Schiemanck, L. Smid, J.L. Stöger, L.G. Visser, J.J.C. de Vries, M.A. Wijngaarden, J.J.M. Geelhoed, A.H.E. Roukens

**Affiliations:** a Department of Cardiology, Leiden University Medical Center, Albinusdreef 2, 2333ZA Leiden, the Netherlands; b Department of Gynaecology, Leiden University Medical Center, the Netherlands; c Department of Intensive Care, Leiden University Medical Center, the Netherlands; d Department of Pulmonary Diseases, Leiden University Medical Center, the Netherlands; e Department of Medical Microbiology, Leiden University Medical Center, the Netherlands; f Department of Infectious Diseases, Leiden University Medical Center, the Netherlands; g Department of Oncology, Leiden University Medical Center, the Netherlands; h Department of Clinical Epidemiology, Leiden University Medical Center, the Netherlands; i Department of Psychiatry, Leiden University Medical Center, the Netherlands; j Department of Rehabilitation Medicine, Leiden University Medical Center, the Netherlands; k Department of Radiology, Leiden University Medical Center, the Netherlands; l Department of Internal Medicine, section Geriatrics, Leiden University Medical Center, Leiden, the Netherlands

**Keywords:** COVID-19, Out-patient clinic, Multidisciplinary follow-up, Cardiopulmonary function, Psychological distress

## Abstract

**Background:**

Short-term follow-up of COVID-19 patients reveals pulmonary dysfunction, myocardial damage and severe psychological distress. Little is known of the burden of these sequelae, and there are no clear recommendations for follow-up of COVID-19 patients.

In this multi-disciplinary evaluation, cardiopulmonary function and psychological impairment after hospitalization for COVID-19 are mapped.

**Methods:**

We evaluated patients at our outpatient clinic 6 weeks after discharge. Cardiopulmonary function was measured by echocardiography, 24-hours ECG monitoring and pulmonary function testing. Psychological adjustment was measured using questionnaires and semi-structured clinical interviews. A comparison was made between patients admitted to the general ward and Intensive care unit (ICU), and between patients with a high versus low functional status.

**Findings:**

Eighty-one patients were included of whom 34 (41%) had been admitted to the ICU. New York Heart Association class II-III was present in 62% of the patients. Left ventricular function was normal in 78% of patients. ICU patients had a lower diffusion capacity (mean difference 12,5% *P* = 0.01), lower forced expiratory volume in one second and forced vital capacity (mean difference 14.9%; *P*<0.001; 15.4%; *P*<0.001; respectively). Risk of depression, anxiety and PTSD were 17%, 5% and 10% respectively and similar for both ICU and non-ICU patients.

**Interpretation:**

Overall, most patients suffered from functional limitations. Dyspnea on exertion was most frequently reported, possibly related to decreased DLCOc. This could be caused by pulmonary fibrosis, which should be investigated in long-term follow-up. In addition, mechanical ventilation, deconditioning, or pulmonary embolism may play an important role.

**Research in context**

**Evidence before this study**

COVID-19 has rapidly emerged as a severe disease with a substantial burden on global healthcare. With more and more patients surviving this disease, also after severe respiratory failure and ICU admission, there is a need for more data on the sequelae of the disease. It has been suggested that cardiac involvement and signs of active myocarditis can be observed on cardiac MRI in almost two-third of patients. Others showed limited abnormalities on additional pulmonary testing, even in patients with residual dyspnea symptoms. In a recent Italian study COVID-19 patients self-report a high risk for anxiety, PTSD and depression. Thus, little is known on the short-term burden of symptoms, residual cardiopulmonary dysfunction and strategies for dedicated follow-up of patients.

**Added value of this study**

In this multidisciplinary evaluation we have demonstrated that there is a diminished pulmonary diffusion capacity, and this is associated with both symptoms and performance score at 6 weeks at the out-patient clinic. Despite a much-expected residual functional impairment, there are no severe cardiopulmonary and psychological abnormalities after hospital admission for COVID-19. Results are comparable to other studies evaluating patients after (severe) respiratory infections. Unlike previously suggested, no severe cardiac dysfunction was observed on echocardiography in our cohort.

**Implications of all the available evidence**

The present manuscript demonstrates that at short term follow-up after COVID-19 many patients do have residual symptoms, mostly dyspnea on exertion. The precise underlying pathophysiological mechanism has yet to be clarified and is likely multifactorial, which implicates that multidisciplinary evaluation of COVID-19 patients is of paramount importance.

Similarly, whether psychological sequelae remain over time needs further follow-up

Alt-text: Unlabelled box

## Introduction

Worldwide, patients who have been admitted to hospital for coronavirus disease 2019 (COVID-19), are currently monitored for (semi-)long term psychological, pulmonary, cardiac or other physical sequelae. Several abnormalities can be expected.

Severe acute respiratory syndrome coronavirus (SARS-CoV) and the Middle East respiratory syndrome coronavirus (MERS-CoV) are known to cause persistent physiological impairment and abnormal radiologic findings consistent with fibrotic lung disease in 30% of those who survived the infection [1,2]. Therefore, it could be that SARS-CoV-2 infection also leads to pulmonary fibrosis in a substantial proportion of post-COVID-19 patients. In addition, researchers have recently reported COVID-19 cardiac involvement via cardiac magnetic resonance imaging 71 days after discharge in almost 80%, and active myocardial inflammation by abnormal native T1 mapping and T2 relaxation times in 60% of 100 recovering patients [3].

Other expected sequelae are hospital acquired muscle weakness, which occurs in 25–50% of the general critical care population. Similarly, persistent fatigue is reported elaborately by COVID-19 patients and might impact rehabilitation. Post-traumatic stress disorder (PTSD), anxiety, depression and mild cognitive impairment are possible disorders which might be expected, especially after intensive care (ICU) admittance [4,5]. In a 2-year follow-up study of ICU patients with acute lung injury, the majority (59%) suffered from anxiety, depression and symptoms of PTSD simultaneously during the entire follow-up [6]. Moreover, risk factors in the general population to develop psychological distress and psychiatric symptoms during a pandemic include presence of chronic/psychiatric illnesses, female gender, younger age, financial loss, inadequate supplies, inadequate information, longer in quarantine, stigmatization (also among health care workers) [7]. These risk factors also apply to COVD-19 patients.

The above-mentioned potential sequelae warrant the need for a multidisciplinary evaluation of COVID-19 survivors. At present, the burden of these sequelae has not been elucidated and little is known on how to organize out-patient follow-up of these patients. Especially in current times with ongoing transmission of SARS-CoV-2, and the onset of a “second wave”, it is of paramount importance to set up a dedicated and efficient follow-up of COVID-19 patients. Therefore, we have monitored patients with confirmed COVID-19 who had been admitted to the general ward or to the Intensive Care Unit at Leiden University Medical Center (585 beds) with a multidisciplinary team, including pulmonary, cardiac, infectious and psychological assessment. The results of this holistic short-term outpatient follow-up may provide guidance for further structured outpatient follow up.

## Methods

### Design and study population

A 6 weeks follow-up study after discharge was conducted to investigate the prognosis of patients surviving COVID-19 who had been admitted for their illness. Patients admitted with PCR-confirmed SARS-CoV-2 infection between March 23rd and June 23rd 2020 were planned for the outpatient clinic after hospital discharge. All adult patients (≥ 18 years) who are living in the Leiden area were eligible. Clinical characteristics at presentation and during hospital admission were collected according to the International Severe Acute Respiratory and emerging Infections Consortium (ISARIC) protocol [8].

### Virus shedding/ inflammatory response

If patients did not have a negative SARS-CoV-2 PCR at discharge, a nasopharyngeal swab for SARS-CoV-2 PCR was collected from patients at the visit 5 weeks after discharge. In addition, C-reactive protein (CRP), lymphocyte count, ferritin, and SARS-CoV-2 IgG (ARCHITECT, Abbott) were measured.

### Cardiac evaluation

Patients were evaluated by a cardiologist 6 weeks after discharge. Cardiac complaints including chest pain, dyspnea and palpitations were assessed. Dyspnea was scored according to the New York Heart Association (NYHA) classification. Resting 12-lead electrocardiogram, 24-hours ECG-monitoring and echocardiography was performed to evaluate cardiovascular manifestations. Left ventricular (LV) systolic function was evaluated by calculating LV ejection fraction (LVEF) using the modified Simpson rule after quantification of the LV end-systolic and end-diastolic volumes from the apical two- and four-chamber view. Right ventricular (RV) function was assessed by measuring tricuspid annular plane excursion in the RV free wall. Troponin T and N-Terminal Pro–B-Type Natriuretic Peptide (NT-proBNP) were determined as markers of cardiac injury and heart failure respectively. Lipid profile was determined to further predict cardiovascular risk profile.

### Pulmonary evaluation

Computed tomography (CT) of the chest was performed upon hospital admission as part of routine clinical care. Suspicion of COVID-19 was assessed according to the COVID-19 Reporting and Data System (CO-RADS) [9]. Additionally, radiologists provided a CT severity score (CTSS), which semi-quantitatively estimates the degree of lung involvement. This entails a 6-point scale per lobe, where 0 equals no involvement, 1 describes <5%, 2 indicates 5–25%, 3 describes 26–50%, 4 equals 51–75%, and 5 indicates >75% involvement, resulting in a total score ranging from 0–25 points. This system was set up in line with a previous method by Chang et al. [10].

Pulmonary function tests were performed at 6 weeks after discharge to assess obstructive and restrictive pulmonary diseases and diffusion disorders. We measured forced expiratory volume in one second (FEV1), forced vital capacity (FVC), the diffusion capacity of the lung for carbon monoxide and corrected for hemoglobin (DLCOc) and the transfer coefficient of the lung for carbon monoxide and corrected for hemoglobin (KCOc)). The Tiffeneau index (Ti) was calculated by dividing the FEV1 by the FVC as a measure of airway obstruction. In addition, forced oscillation technique (FOT) was used as a tool to measure respiratory resistance (Rrs) as a sign of airway obstruction at 5, 11 and 19 Hz during 60 s of tidal breathing.

### Psychological evaluation in patients and caregivers

All patients were invited to fill out self-report screening questionnaires assessing psychological and cognitive symptoms. Patients’ caregivers/partners were invited to act as a proxy informant on the patients’ cognitive functioning. Furthermore, patients were followed-up by a clinical psychologist who evaluated psychological adjustment in a semi-structured clinical interview. Anxiety, depression, posttraumatic stress symptoms and cognitive functioning were measured using respectively the GAD-7 (Generalized Anxiety Disorder Scale, cut-off score for moderate-severe anxiety of ≥ 10) [11]. PHQ-9 (Patient Health Questionnaire 9, cut-off score for moderate-severe depression ≥ 10) [12], PCL_5 (PTSD Checklist for the DSM 5, cut-off score for PTSD ≥ 38) [13]. the CFQ-25 (Cognitive Failures Questionnaire, cut-off score for cognitive impairments ≥ 31) [14] and an adapted version of the IQ-CODE-N (Informant Questionnaire on Cognitive Functioning in the Elderly, cut-off score for cognitive decline following COVID-19 > 3.31) [15]. Baseline characteristics of responders and non-responders were compared to rule out responder-bias. A more detailed description of the used questionnaires is provided in the supplements.

### Post-COVID-19 functional status (PCFS) score

Recently, the concept of a ordinal scale for the assessment of patient-relevant functional limitations has been proposed for COVID-19 patients [16]. In each patient, the PCFS score was assigned based on the medical charts by two independent observers. In brief, grade 0 reflects unlimited daily functioning. In grade 1 symptoms, pain or anxiety are present but have no effect on activities. In grade 2, daily activities are performed at a lower intensity. Grade 3 patients are forced to structurally modify their activities due to symptoms, pain or anxiety. Finally, grade 4 patients require assistance with activities of daily living because of severe functional limitations. For the present analysis, we have stratified patients according to low functional status (i.e. PFCS grade ≥3) and high functional status (i.e. PCFS grade <3).

### Statistical analysis

Descriptive statistics were used for all variables. Continuous variables were reported as means with standard deviations or median and interquartile ranges; categorical variables were reported as numbers with percentages. The independent Sample T-test was used to compare continuous variables, and a Chi-Square test was used for categorical variables. Results of patients who had been admitted to the ICU versus patients who had only been admitted to the general ward, were compared. Furthermore, this was done for patients with a high (i.e. ≥3) versus low (i.e. <3) PCFS-score. Patients with a low versus a high NYHA class were compared to elucidate the possible influence of cardiopulmonary function on functional status. All statistical analyses were performed using IBM SPSS version 25.0

### Reporting and ethics

The study was approved by the hospital's ethical review board (Ethical Committee for COVID-19 related research at the LUMC, protocol number 2020–059) Research data were pseudonymized and securely stored, according to the General Data Protection Regulation. Informed consent was obtained by an opt-out procedure. All patients admitted to the hospital were given a letter which stated that their data could be used for research purposes, and that they could opt out if they disagreed. None of the admitted patients have declined consent.

### Role of the funding

There was no external funding for this analysis.

## Results

### Baseline result

There were initially 175 patients with COVID-19 admitted in the LUMC. Of these, 35 (20%) died during admission and 2 were transferred to a hospice for palliative care. None of remaining patients died between discharge and first visit at the outpatient clinic. Thirty-six patients were transferred from another region and were therefore not scheduled for follow-up at our clinic. Of the remaining patients (*n* = 102), 7 (7%) patients declined outpatient follow-up because of absence of symptoms. Three patients were admitted during follow-up for another condition and 10 patients were not scheduled due to administrative errors. Finally, 81 patients were included in the present study. (data not show in Tables) Baseline characteristics are depicted in Table 1. Of the total population of 81 patients, 51 (63%) were male, mean age was 61±13 years, with a range of 27–88 years. Mean Body Mass Index was 27·8 ± 4·5 kg/m^2^.There were 23 (28%) patients with pre-existent cardiovascular disease, and 18 patients with an pre-existent pulmonary disease. The patients with an underlying pulmonary disease included 12 patients (15%) with asthma, 3 patients with COPD (4%), 1 patient with a pulmonary embolism (1%), and 2 patients with OSAS (3%).

**Table 1:** Baseline characteristics.

Characteristic (*N* = 81)	
Age	60·8 ± 13
Gender (number of males)	51 (63%)
BMI	27·8 ± 4·5
Cardiovascular disease	23 (28%)
Heart failure	1 (1%)
Atrial fibrillation	4 (5%)
Valvular heart disease	5 (6%)
CABG/PCI	10 (12%)
Myocardial infarction	3 (4%)
CVA/TIA	8 (10%)
PVD	2 (2%)
Chronic Kidney Injury	9 (11%)
Hypertension	28 (34%)
Hypercholesteremia	22 (27%)
Diabetes	19 (23%)
Family history of CVD	10 (12%)
Smoking	9 (11%)
Alcohol	19 (23%)
Lung disease	17 (21%)
Asthma	12 (15%)
COPD	3 (4%)
Pulmonary embolism	1 (1%)
OSAS	2 (3%)
Immunodeficiency	6 (7%)
Kidney transplantation	3 (4%)
Rheumatoid arthritis (MTx)	2 (2%)
HIV	1 (1%)
Medication (pre-admission)	
BB/CCB (Non-dihydropyridin)	18 (22%)
Anti-HT	32 (39%)
Statin	25 (31%)
Diabetes	14 (17%)
Platelets inhibition	16 (20%)
Anti-coagulation	6 (7%)
Treatment	
Remdesivir (number)	23 (28%)
Remdesivir (average, days)	6·2 ± 2·3
(Hydroxy)chloroquin (number)	48 (59%)
(Hydroxy)chloroquin (average, days)	4·4 ± 1·5
Steroids (number)	3 (4%)
Steroids (average, days)	15·8 ± 12·3

Details of the patient population at baseline and medical treatment for COVID −19. Data are presented a number (percentages) of mean ± standard deviation.

Abbreviations: BB, beta blocker BMI: body mass index, CABG: coronary artery bypass graft, CCB: calcium channel blocker, COPD: chronic obstructive pulmonary disease, CVA: cerebrovascular accident, CVD: cardiovascular disease, HIV: human immunodeficiency virus, HT; hypertension MTx, methotrexate, OSAS: obstructive sleep apnea syndrome, PCI: percutaneous coronary intervention, PVD: Peripheral vascular disease, TIA: transient ischemic attack.

### Overall population

Six weeks after discharge, 14% of the patients reported complaints of chest pain and NYHA class II-III was present in 62% of the patients. Twenty-six patients (32%) had a PCFS score of 0 or 1, corresponding with very limited functional limitations. Eighteen patients (22%) had a score of 2, equal to daily activities at a lower intensity. The remaining 37 patients (45%) had considerable functional impairment with a score of 3 or 4. Echocardiography revealed normal LV and RV function in most patients (78% and 84% respectively). In only 2 of the 15 patients with LV dysfunction (LVEF <52%), this was a known pre-existing condition. Mean high sensitive troponin-T and NT-ProBNP at follow-up were low, 11±11 ng/L and 190±435 ng/L, respectively.(Supplements Table 3). During 24-hours ECG-monitoring no rhythm disturbances were noted except for 2 patients with known atrial fibrillation. Mean heart rate was 78±17 beats/per minute. There were no differences in mean heart rate between the different groups.

The baseline computed tomographic (CT) imaging showed peripheral lung ground-glass opacities with or without organizing pneumonia in most of the patients. In the total study group, the mean CO-RADS was 5.3 (SD 0.6) and the mean CTSS was 12.7% (SD 4.9%). Pulmonary function tests demonstrated a mean FEV1 of 3·03±0.88 L/s (94.5% of predicted ± 19·8%), the mean FVC was 3·82±1.14 L/s (92·5% of predicted ±20.9%), the mean Ti was 101±14% and the mean DLCOc was 78±16% of predicted (Table 4). Interestingly, none of the patients showed an obstructive pulmonary function as measured by a Ti (101±14%) and additionally supported by normal FOT measurement in all patients (data not shown)

Out of 81 eligible patients, 4 were unable to fill out the psychological questionnaires (language difficulties *n* = 3; pre-existing cognitive impairment *n* = 1). Of the remaining 77 patients, 59 (77%) completed the questionnaires. No significant differences were found between responders and non-responders in terms of age (*P* = 0.646), gender (*P* = 0.453), ICU admission (*P* = 0.250) or PCFS-score (*P* = 0.697). The IQ-CODE-N was completed by 38 (64%) caregivers/partners. (data not show in Tables) Elevated symptoms of depression, anxiety or PTSD occurred in respectively 17%, 5% and 10% of patients and 40% of these patients reported previous or current mental health treatment. Cognitive impairments were reported by approximately one quarter of patients.

At admission, more than 60% had a lymphopenia (defined as < 1 × 10^9^/L), and more than 90% had an elevated ferritin and CRP (Supplements Table 3). These inflammatory parameters did not differ between the ICU and non-ICU patients, or those with a PCFS < 3 of ≥ 3 (supplements Table 4). These parameters, except ferritin, normalized in all patients after discharge. All patients at the outpatient clinic had a positive SARS-CoV-2 IgG response. In 62 of the 81 patients SARS-CoV-2 in the PCR of a nasopharyngeal swab was assessed at 5 weeks, 61 (98%) had a negative test-result. (data not show in Tables)

### ICU admission

Thirty-four patients (41%) had been admitted to the ICU, of whom 33 required mechanical ventilation (Table 2). All patients were stepped down to a normal ward before discharge. One patient received high dose methylprednisolone for 30 days. Mean duration of ICU admission was 20 ± 13days, and on average patients were on mechanical ventilation for 16±10 days. There were no significant differences in baseline demographics, cardiovascular risk factors and relevant co-morbidities between patients who were admitted to the ICU compared with patients admitted to the general ward. (Supplement Table 1). Twenty-two of the 34 (64%) ICU patients had a low functional status (PCFS≥3) at 6 weeks compared to 15 of the 47 (32%) non-ICU patients (*P* = 0·003). Patients admitted to the ICU also had a higher NYHA class. However, deterioration of LV and RV function was not related to ICU admission. We did find a correlation between of DLCOc, FEV1 and FVC with ICU admission (Table 4). DLCOc value was significantly lower in the ICU group versus the non-ICU group (mean difference 12·5% *P* = 0·01). Also, FEV1 and FVC were significantly lower in de ICU group than in the non-ICU group (mean difference FEV1 14·9%; *P*<0·001; mean difference FVC 15·4%; *P*<0.001; respectively). Even when diffusion capacity was corrected for alveolar volume, KCOc, was not significantly higher in the ICU group versus the non-ICU group (mean difference 1·76%; *P* = 0·652). In the ICU group a higher incidence of pulmonary embolism was observed during admission (12 (35%) vs 6 (13%), *P* = 0·062). Additionally, CT Severity index was significantly higher in the ICU group (15·6 ± 5·2) compared to ward patients (10·8 ± 3·6) (*P*<0·001). Of interest, no significant differences were found in psychological and cognitive functioning between ICU and non-ICU patients.

**Table 2:** Admission details for the overall population and stratified according to ICU admission.

Characteristic (*N* = 81)	Total	No ICU Admission (*N* = 47)	ICU Admission (*N* = 34)	P-value
**Chest CT**				
CORADS (*n* = 73)	5·3 ± 0·6	5·2 ± 0·52	5·6 ± 0·6	**0·004**
CT Severity score (*N* = 78)	12·7 ± 4·9	10·8 ± 3·6	15·6 ± 5·2	**<0·001**
**Admission details**				
Duration symptoms admission (average, days)	9·7 ± 4·3	10·5 ± 4·8	8·7 ± 3·2	0·064
Duration hospitalization (average, days)	17·1 ± 15·2	7·45±4·1	30·5 ± 14·8	**<0·001**
Admission ICU (number)	34 (42%)			
Durations admission ICU (average, days)	19·8 ± 12·5	NA	19·8 ± 12·5	NA
Mechanical ventilation (number)	33 (41%)	NA	33 (97%)	NA
Mechanical ventilation (duration)	15·9 ± 10·3	NA	15·9 ± 10·3	NA
Thromboembolic complications (number)	18 (22%)	6 (13%)	12 (35%)	0·062
**Discharge**				
Discharged home (number)	51 (62%)	36 (77%)	15 (44%)	**0·003**

Details of the admission for COVID-19, including CT characteristics, and admission duration, thromboembolic complications and discharge characteristics. Data are depicted for the overall population and stratified according to admission to the ICU or general ward. Data are presented a number (percentages) of mean ± standard deviation.

Abbreviations: CT: computed tomography, CO-RADS: COVID-19 imaging reporting and data system ICU: intensive care unit.

### Functional status

A comparison was made between patients with a high vs low overall functional status. Baseline characteristics, and risk factors were evenly distributed between both groups (Supplement Table 2). Patients with a low functional status had been admitted to the hospital for a significantly longer period and more often needed mechanical ventilation (Table 3). By definition, patients with a low functional status had a higher NYHA class at 6 weeks, but there was no association with cardiac dysfunction (Table 5). Whereas, in patients with a lower functional status a lower DLCOc (mean difference 6·6%; *P* = 0·075), lower FEV1 and FVC (mean difference FEV1 12·8%; *P* = 0·003; mean difference FVC 14·6%; *P*<0.001; respectively) were found (Table 5). Also, in the lower functional status group the KCOc was higher compared to a high functional status, but not significant (mean difference 3·62%; *P* = 0.329).

**Table 3:** Admission details stratified according to PCFS-score.

Characteristic (*N* = 81)	PCFS <3 (*N* = 44)	PCFS ≥3 (*N* = 37)	P-value
**Chest CT**			
CORADS (*n* = 73)	5·3 ± 0·6	5·4 ± 0·7	0·656
CT Severity score (*N* = 78)	11·8 ± 3·8	13·8 ± 5·9	0·081
**Admission details**			
Duration symptoms admission (average, days)	11·1 ± 4·4	8·8 ± 3·5	**0·001**
Duration hospitalization (average, days)	11·7 ± 9·7	23·5 ± 18·0	**<0·001**
Admission ICU (number)	12 (27%)	22 (59%)	**0·003**
Durations admission ICU (average, days)	14·3 ± 9·0	18·9 ± 12·6	0·127
Mechanical ventilation (number)	12 (27%)	21 (57%)	**0·007**
Mechanical ventilation (duration)	12·6 ± 6·8	17·7 ± 11·5	0·134
Thromboembolic complications (number)	7 (16%)	11 (30%)	0·136
**Discharge**			
Discharged home (number)	33 (75%)	18 (49%)	**0·014**

Details of the admission for COVID-19, including CT characteristics, and admission duration, thromboembolic complications and discharge characteristics. Data are stratified according to a low and high functional status based on the Post-COVID-19 Functional Status (PCFS)-score. Data are presented a number (percentages) of mean ± standard deviation.

Abbreviations: CT: computed tomography, CO-RADS: COVID-19 imaging reporting and data system, ICU: intensive care unit.

**Table 4:** Outpatient clinic evaluation for the overall population and stratified according to ICU admission.

Characteristic (*N* = 81)	Total	No ICU Admission (*N* = 47)	ICU Admission (*N* = 34)	P-value
**Complaints**				
Chest pain				0·374
Non-anginal	11 (14%)	4 (9%)	7 (21%)	
Atypical	3 (4%)	2 (4%)	1 (3%)	
Typical	1 (1%)	1 (2%)	0(0%)	
None	66 (82%)	40 (91%)	26 (76%)	
NYHA class				**0·017**
1	31 ((38%)	24 (51%)	11 (32%)	
2	37 (46%)	20 (43%)	13 (38%)	
3	13 (16%)	3 (6%)	10 (29%)	
4	0(0%)	0(0%)	0(0%)	
Palpitations	12 (15%)	7 (15%)	5 (15%)	0·981
**Mental health***n* = 59				
Psychiatric morbidities				
Anxiety (GAD-7 ≥ 10),	3 (5%)	2 (6%)	1 (4%)	0·418
Depression (PHQ-9 ≥ 10),	10 (17%)	6(19%)	4 (15%)	0·736
PTSS (PCL-5 ≥ 38),	5 (10%)	3(11%)	2 (9%)	0·984
Psychiatric morbidities	10 (17%)	6 (18%)	4 (15%)	0·776
Cognitive functioning				
Cognitive Failures (CFQ-25 > 31·8),	13(27%)	7(25%)	6 (30%)	0·701
Partners (I-Code-16 ≥ 3·31),	10 (26%)	5(25%)	5(28%)	0·085
**Echocardiography**				
LV function (LVEF%)	56·3 ± 6·0	56·7 ± 5·9	55·7 ± 5·9	0·451
>52	63 (78%)	38 (81%)	25 (74%)	0·337
41–51	14 (17%)	6 (13%)	8 (24%)	
30–40	1 (1%)	1 (2%)	0(0%)	
<30	0(0%)	0(0%)	0(0%)	
RV function TAPSE	21±3·2	21·0 ± 3·4	20·8 ± 2·9	0·701
TAPSE >17mm	68 (84%)	38 (81%)	30 (81%)	0·721
TAPSE <17mm	8 (10%)	5 (11%)	3 (9%)	
**Spirometry**				
FEV1 L/s	3·03±0·88	3·16±0·87	2·83 ± 0·88	0·111
FEV1 in%	94·5 ± 19·8	100·8 ± 18·1	85·8 ± 19·0	**0·001**
FVC L/s	3·82 ± 1·14	4·02±1·07	3·55 ± 1·20	0·072
FVC in%	92·5 ± 20·9	99·0 ± 18·5	83·5 ± 20·9	**0·001**
Ti in%	101·0 ± 13·9	101·6 ± 10·6	100·1 ± 17·6	0·661
DLCOc in%	78·4 ± 15·8	83·5 ± 14·6	71·1 ± 14·7	**0·001**

Overview of the outpatient characteristics, including overall symptoms, psychometric evaluation and cardiopulmonary function. Data are depicted for the overall population and stratified according to admission to the ICU or general ward. Data are presented a number (percentages) of mean ± standard deviation.

Abbreviations: CFQ: cognitive failure questionnaire, DLCOc: diffusion capacity of the lung for carbon monoxide and corrected for hemoglobin, FEV1: forced expiratory volume in one second, FVC: forced vital capacity, GAD: generalized anxiety disorder scale, I-code: informant questionnaire on cognitive functioning in the elderly, LV: left ventricular, LVEF: left ventricular ejection fraction, NYHA: New York Heart Association, PCL: post-traumatic stress disorder checklist PHQ: patient health questionnaire, RV: right ventricular; TAPSE: tricuspid annular plane systolic excursion, Ti: Tiffeneau index.

**Table 5:** Outpatient clinic evaluation stratified according to PCFS-score.

Characteristic (*N* = 81)	PCFS <3 (*N* = 44)	PCFS ≥3 (*N* = 37)	P-value
**Complaints**			
Chest pain			**0·042**
Non-anginal	2 (5%)	9 (24%)	
Atypical	2 (5%)	1 (3%)	
Typical	0	1 (3%)	
None	40 (91%)	26 (70%)	
NYHA class			**0·025**
1	21 (48%)	10 (27%)	
2	12 (28%)	17 (46%)	
3	3 (7%)	10 (27%)	
4	0 (0%)	0(0%)	
Palpitations	5 (11%)	7 (19%)	0·340
**Mental health***n* = 59			
Psychiatric morbidities			
Anxiety (GAD-7 ≥ 10),	1 (3%)	2 (8%)	0·418
Depression (PHQ-9 ≥ 10),	1 (3%)	9 (35%)	**0·002**
PTSS (PCL-5 ≥ 38),	1 (4%)	4 (16%)	0·157
Psychiatric morbidities	1 (3%)	9 (35%)	0·001
Cognitive functioning			
Cognitive Failures (CFQ-25 > 31·8),	5 (19%)	8 36%)	0·183
Partners (I-Code-16 ≥ 3·31),	1 (5%)	9 (53%)	**0·001**
**Echocardiography**			
LV function (LVEF%)	56·3 ± 6·3	53·3 ± 5·5	0·979
>52	34 (77%)	29 (78%)	0·647
41–51	8 (18%)	6 (16%)	
30–40	1 (2%)	0(0%)	
<30	0(0%)	0(0%)	
RV function TAPSE	20·7 ± 3·2	21·1 ± 3·1	0·554
TAPSE >17mm	37 (84%)	31 (84%)	0·663
TAPSE <17mm	5 (11%)	3 (8%)	
**Spirometry**			
FEV1 L/s	3·28 ± 0·92	2·70± (0·72)	**0·02**
FEV1 in%	100·2 ± 20·5	87·4 ± 16·5	**0·03**
FVC L/s	4·21 ± 1·21	3·33 ± 0·85	**0·00**
FVC in%	99·0 ± 20·8	84·4 ± 18·1	**0·01**
Ti in%	78·8 ± 7·5	80·7 ± 7·3	0·271
DLCOc in%	81·2 ± 15·8	74·6 ± 15·2	0·074

Overview of the outpatient characteristics, including overall symptoms, psychometric evaluation and cardiopulmonary function. Data are stratified according to a low and high functional status based on the Post-COVID-19 Functional Status (PCFS)-score. Data are presented a number (percentages) of mean ± standard deviation.

Abbreviations: CFQ: cognitive failure questionnaire, DLCOc: diffusion capacity of the lung for carbon monoxide and corrected for, FEV1: forced expiratory volume in one second, FVC: forced vital capacity, GAD: generalized anxiety disorder scale, I-code: informant questionnaire on cognitive functioning in the elderly, LV: left ventricular, LVEF: left ventricular ejection fraction, NYHA: New York Heart Association, PCL: post-traumatic stress disorder checklist PHQ: patient health questionnaire, RV: right ventricular; TAPSE: tricuspid annular plane systolic excursion, Ti: Tiffeneau index.

**Fig. 1:**
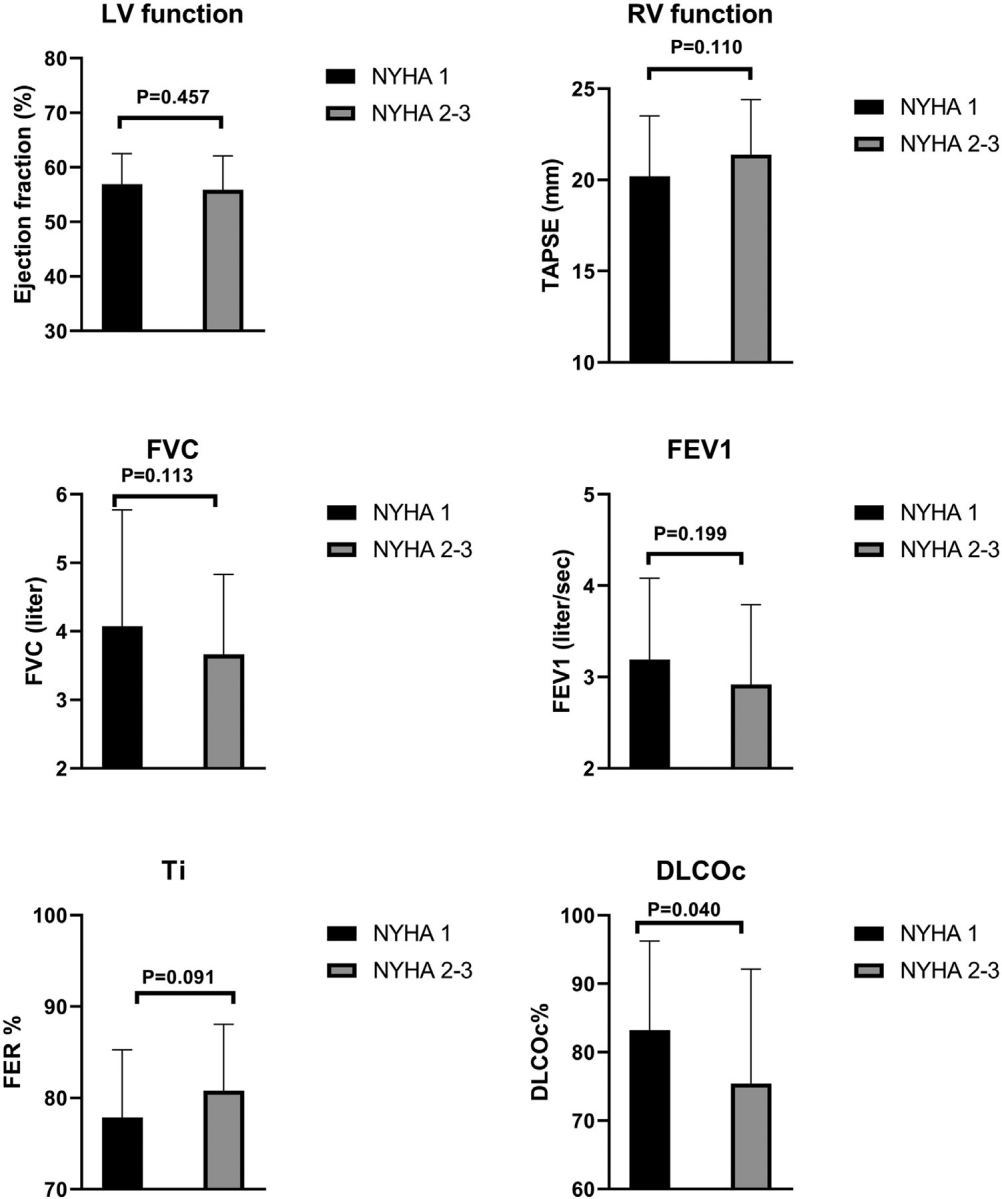
Relation between NYHA class and cardiopulmonary function Bar-graph demonstrating cardiopulmonary function stratified according to NYHA-class. A comparison is made between patients with and without dyspnea on exertion. Both left and right ventricular function and pulmonary function tests are analyzed.

Comparing NYHA class with cardiopulmonary function revealed no significant differences except for DLCOc values (Fig. 1). Patients with higher NYHA class (2 – 3) had significantly lower DLCOc values (75 ± 17% vs 83 ± 13%, *P* = 0.040). There was a trend towards a lower FVC, FEV1 in patients with a higher NYHA-class. Patients in the subgroup with a low functional status were more likely to be at risk of depression and suffer from cognitive problems (as indicated by their caregiver/partner) as compared to the group of patients with a better functional status, i.e. low score on the PCFS (see Table 5). There was a good correlation between the PCFS-score and self-reported psychological functioning (Supplement Table 5).

## Discussion

In the present study, the majority of COVID-19 patients had residual symptoms at the outpatient clinic 6 weeks after discharge, mainly dyspnea on exertion as assessed by NYHA class. Patients showed relatively good psychological adjustment and limited prevalence of depression, anxiety or PTSD symptoms (ranging from 5–17%). Cardiac function was normal in majority of the patients and LV dysfunction was not related to functional status assessed by NYHA class or PCFS score. However, we found a lower pulmonary diffusion capacity correlated with functional status, dyspnea. In patients admitted to the ICU a lower diffusion capacity was observed. Our results are very comparable to a recent UK cohort study by Arnold et al. in which 110 COVID-19 patients were evaluated at an outpatient clinic [17]. In their cohort 74% of patients had residual symptoms, mainly dyspnea and fatigue. Only 10% of patients had restrictive spirometry. Cardiac function was not assessed in that study. Interestingly, in contrast to our findings, no relation was observed between symptoms and clinical test results. Similar results have previously been found in patients surviving an Influenza A (H1N1)-associated ARDS [18]. In these patients, at one year post-ICU discharge, minor lung disabilities with diminished diffusion was observed compared to a sex- and age-matched general population group.

It remains to be established how frequently primary cardiac involvement (i.e. myocardial fibrosis or myocarditis) occurs, or that troponin release is more related to secondary damage due to extra-cardiac mechanisms such as cytokines, hypoxia, pulmonary embolism and sepsis [19]. The present finding supports the latter, given that there are few patients with significant cardiac dysfunction after 6 weeks. In contrast, a recent study analyzing cardiac function by MRI in post-COVID-19 patients, demonstrated extensive myocardial damage after COVID-19, although there was no data on prior cardiac dysfunction in these patients [3]. Evidently, an increased BMI, obesity and diabetes can result in alterations in cardiac function [20] Taking into consideration the augmented cardiovascular risk (marked by the increased BMI, distorted lipid profile, high prevalence of diabetes and hypertension) in this population, subtle LV or RV function may have been pre-existent and is not caused by COVID-19. In addition, previous studies in severe influenza infection have shown that right and left ventricular dysfunction can be observed in a significant percentage of patients and this was not related to prognosis [21]. In addition, during follow-up in the current study no significant arrhythmias were observed on 24 h ECG-monitoring, which offers no immediate support to the hypothesis that increased out-of-hospital cardiac arrests during the pandemic are caused by COVID-19 [14].

Pulmonary function testing showed a trend towards a more restrictive pulmonary function in combination with a lower diffusion capacity; this was mostly present both in ICU patients and in patients with lower functional status. The lower DLCOc could be partly explained by the lower FVC, however since the KCOc was also lower in the IC group and the lower functional status group, it could be a true decreased diffusion capacity. Furthermore, the higher incidence of pulmonary embolism in the ICU group could be related to the lower diffusion capacity. The poor oxygenation at presentation, requiring ventilation and oxygen administration, in COVID-19 might be directly related to decreased diffusion capacity due to parenchymal destruction as well as increased alveolar-capillary distances. This was illustrated by the fact that at presentation most of the patients showed peripheral lung ground-glass opacities on computed tomographic (CT) imaging of the chest with a high severity index. Regeneration of edema and thrombosis is most likely reversible, whereas interstitial fibrosis, might be irreversible. Previous studies on survivors of acute lung injury (ALI and ARDS) [22] have shown variable degrees of residual abnormalities in pulmonary function, exercise capacity, and impairment in health-related quality of life after 1-year follow-up. In addition, the exercise capacity and health status of SARS survivors in 2003 was considerably lower than that of a healthy population after 6 months follow-up. Significant impairment in surface area for gas exchange was noted in 15.5% of survivors [1]. Recent data suggest that COVID-19 related ARDS has similar characteristics compared to non-COVID-19 ARDS [23]. Long term outcomes in survivors of epidemic Influenza A (H7N9) virus infection also suggest long-term lung disability and psychological impairment persisting at 2 years after discharge from the hospital [24]. We know from recent studies that histologically, COVID-19 is characterized by a combination of the early phase of acute respiratory distress syndrome (ARDS) with diffuse alveolar damage in combination with pulmonary-vascular changes [25,26]. Only few cases of rapid onset extensive pulmonary fibrosis following COVID-19 have been reported so far.

A small group of patients reported clinically elevated levels of psychological distress 6 weeks after discharge from hospital, and there were no significant differences between ICU and general ward patients. The percentage of patients meeting the criteria for depression (17%) is comparable to that reported by a meta-analysis on SARS, MERS & COVID-19 patients) [27], and in line with prevalence rates observed in other patient groups facing a serious illness or event [28]. Prevalence rates for anxiety (5%) and PTSD-symptoms (10%) are lower than previously observed in coronavirus patients [5,27,29] and also somewhat lower than those seen in other patient populations [30]. Similarly, approximately one quarter of both ICU and ward patients suffered from cognitive impairments, which is lower than observed previously in critical illness survivors [4]. Possibly, cultural differences and health care system characteristics may play a role; the UK study by Arnold and colleagues also reported relatively good psychological adjustment in the vast majority of patients [17].

Self-report symptom questionnaires – although commonly used for screening purposes – have been criticized for being inaccurate [31]. In this study we therefore used self-report symptom questionnaires followed-up by semi-structured clinical interviews to evaluate level of psychological adjustment. In line with the findings from the screening questionnaires, follow-up psychological treatment was indicated in only a handful of patients. This is similar to findings from the UK study by Arnold and colleagues, which reported that only 5 out of 110 COVID-19 patients had been referred to specialist psychological services [17]. How psychological adjustment and cognitive functioning develop over time should be determined during follow-up. To prevent (worsening of) psychiatric symptoms, attention should be prioritized to the vulnerable groups within the COVID-population, such as those suffering from a chronic/psychiatric illness, women, youngsters and stigmatized groups (including healthcare workers). Besides monitoring, remote mental health services can be offered in the form of online (video) consultations and (guided) eHealth interventions. Healthy lifestyle behavior interventions such as increasing physical and personally meaningful activity, or reducing frequency of exposure to social media/news concerning COVID-19 also can help to prevent worsening of symptoms [7].

Several methodological aspects of our study warrant comment. First, obviously our results only apply to surviving patients who had been admitted, meaning that the findings we report here may not apply to those who died during admission of patients with mild symptoms who had not been admitted. It may very well be that for instance cardiac function deteriorates more severely in COVID-19 non-survivors. Moreover, due to multiple reasons only less than 50% of the patients originally admitted to the hospital formed eventually the study population, which could have introduced some selection bias. Second, none of our comparisons between ICU and non-ICU patients were adjusted for confounding, since numbers were small, yet baseline characteristics between patients with COVID-19 admitted to ICU and the general ward were comparable, which leaves less room for confounding. Finally, for most of the outcome parameters, there were no prior measurements available. For this reason, it is not known whether cardiopulmonary dysfunction was already pre-existent, leading to an overestimation of the occurrence and of the sequelae. It should be noted that the present cohort is relatively small, this could have caused type II error and could have cause a lack of power for the statistical results

Several conclusions can be drawn from the present short-term follow-up study of COVID-19. Importantly, overall, most patients endure mild to moderate functional and psychological limiations. Dyspnea is the most frequently reported symptom, with two-thirds of patients functioning at NYHA II-III, however this was not related to LV or RV dysfunction. A decreased diffusion capacity was observed in a large number of patients, predominantly patients admitted to the ICU, and this correlated with functional limitations at short term follow-up. It is unclear whether the increased NYHA-class and PCFS-score will improve over time. This is likely to be dependent on the underlying mechanism, for example due to pulmonary fibrosis or chronic pulmonary embolism. Therefore, extended multidisciplinary follow-up including chest CT and pulmonary function testing if symptoms persist is warranted. Similarly, patients reporting elevated risk of psychological disturbances should be closely monitored.

## Declaration of Competing Interest

There is no conflict of interest for the present manuscript.

## Appendix Supplementary materials

Image, application 1

Image, application 2
