# Supplementary material for: Short-term outpatient follow-up of COVID-19 patients: A multidisciplinary approach
Source: eClinicalMedicine. 2021 Jan 28;32:100731. doi: 10.1016/j.eclinm.2021.100731 (PMC7843037; doi:10.1016/j.eclinm.2021.100731)
Supplement: Supplementary file 1 [file mmc1.docx]

**Supplementary Table 1. Baseline variables stratified according to ICU admission.**

Details of the patient population at baseline and medical treatment for COVID -19 stratified according to admission to the ICU or general ward. Data are presented a number (percentages) of mean ± standard deviation

| **Characteristic (N=81)** | **No ICU Admission**  **(N=47)** | **ICU Admission (N=34)** | **P-value** |
| --- | --- | --- | --- |
| Age | 63·4±13·8 | 57·3±11·1 | 0·038 |
| Gender (number of males) | 30 (64%) | 21 (62%) | 0·849 |
| BMI | 27·9±4·3 | 27·9±4·8 | 0·953 |
| **Cardiovascular disease** | 16 (34%) | 7 (21%) | 0·185 |
| Heart failure | 1 (2%) | 0(0%) | 0·392 |
| Atrial fibrillation | 4 (9%) | 0(0%) | 0·081 |
| Valvular heart disease | 4(9%) | 1 (3%) | 0·304 |
| CABG/PCI | 6 (13%) | 4 (12%) | 0·892 |
| Myocardial infarction | 2 (4%) | 1 (3%) | 0·757 |
| CVA/TIA | 5 (11%) | 3 (9%) | 0·787 |
| PVD | 1 (2%) | 1 (3%) | 0·816 |
| Chronic Kidney Injury | 5 (11%) | 4 (12%) | 0·874 |
| Hypertension | 18 (38%) | 10 (29%) | 0·407 |
| Hypercholesteremia | 13 (28%) | 9 (26%) | 0·905 |
| Diabetes | 8 (17%) | 11 (32%) | 0·108 |
| Family history of CVD | 5 (11%) | 5 (15%) | 0·583 |
| Smoking | 3 (6%) | 6 (18%) | 0·111 |
| Alcohol | 10 (21%) | 9 (26%) | 0·586 |
| **Lung disease** | 7 (15%) | 10 (29%) | 0·113 |
| Asthma | 4(9%) | 8 (24%) |  |
| COPD | 3 (6%) | 0 (0%) |  |
| Pulmonary embolism | 0(0%) | 1 (3%) |  |
| OSAS | 0(0%) | 2 (6%) |  |
| **Immunodeficiency** | 5 (11%) | 1 (3%) | 0·192 |
| Kidney transplantation | 2 (4%) | 1 (3%) |  |
| Rheumatoid arthritis (MTx) | 2 (4%) | 0 (0%) |  |
| HIV | 1 (2%) | 0 (0%) |  |
| **Medication (pre-admission)** |  |  |  |
| BB/CCB (Non-dihydropyran) | 11 (23% | 7 (21%) | 0·764 |
| Anti -hypertensive drugs | 16(34%) | 16 (47%) | 0·237 |
| Statin | 15 (32%) | 10 (29%) | 0·810 |
| Diabetes | 5 (11%) | 9 (26%) | 0·063 |
| Platelets inhibition | 9 (19%) | 7(21%) | 0·872 |
| Anti-coagulation | 4(9%) | 2 (6%) | 0·656 |

Abbreviations: BB, beta blocker BMI: body mass index, CABG: coronary artery bypass graft, CCB: calcium channel blocker, COPD: chronic obstructive pulmonary disease, CVA: cerebrovascular accident, CVD: cardiovascular disease, HIV: human immunodeficiency virus, HT; hypertension MTx, methotrexate, OSAS: obstructive sleep apnea syndrome, PCI: percutaneous coronary intervention, PVD: Peripheral vascular disease, TIA: transient ischemic attack

| **Characteristic (N=81)** | **PCFS <3 (N=44)** | **PCFS ≥3 (N=37)** | **P-value** |
| --- | --- | --- | --- |
| Age | 61·5±12·9 | 60·0±13·4 | 0·611 |
| Gender (number of males) | 30 (68%) | 21 (57%) | 0·289 |
| BMI | 27·6±4·1 | 28·3±5·0 | 0·535 |
| **Cardiovascular disease** | 12 (27%) | 11 (30%) | 0·807 |
| Heart failure | 0(0%) | 1 (3%) | 0·356 |
| Atrial fibrillation | 3 (7%) | 1(3%) | 0·394 |
| Valvular heart disease | 3 (7%) | 2 (5%) | 0·792 |
| CABG/PCI | 4 (9%) | 6 (16%) | 0·332 |
| Myocardial infarction | 3(7%) | 0(0%) | 0·106 |
| CVA/TIA | 3(7%) | 5 (14%) | 0·314 |
| PVD | 0(0%) | 2 (5%) | 0·118 |
| Chronic Kidney Injury | 6 (14%) | 3 (85) | 0·430 |
| Hypertension | 17 (39%) | 11 (30%) | 0·401 |
| Hypercholesteremia | 11 (25%) | 11 (30%) | 0·634 |
| Diabetes | 7 (16%) | 12 (32%) | 0·080 |
| Family history of CVD | 6 (14%) | 4 (11%) | 0·700 |
| Smoking | 5 (11%) | 4 (11%) | 0·937 |
| Alcohol | 13 (30%) | 6 (16%) | 0·158 |
| **Lung disease** | 7 (165) | 10 (27% | 0·221 |
| Asthma | 6 (14%) | 6 (16%) |  |
| COPD | 1 (2%) | 2 (5%) |  |
| Pulmonary embolism | 0(0%) | 1(3%) |  |
| OSAS | 0(0%) | 2 (5%) |  |
| **Immunodeficiency** | 2(5%) | 4 (11%) | 0·283 |
| Kidney transplantation | 1 (2%) | 2 (5%) |  |
| Rheumatoid arthritis (MTx) | 1 (2%) | 1(3%) |  |
| HIV | 0(0%) | 1(3%) |  |
| **Medication (pre-admission)** |  |  |  |
| BB/CCB (Non-dihydropyran) | 8 (18%) | 10 (27%) | 0·340 |
| Anti -hypertensive drugs | 15 (34%) | 17 (46%) | 0·277 |
| Statin | 13 (30%) | 12 (32%) | 0·779 |
| Diabetes | 4 (9%) | 10 (27%) | **0·033** |
| Platelets inhibition | 7 (16%) | 9(24%) | 0·343 |
| Anti-coagulation | 3(7%) | 3 (8%) | 0·825 |

**Supplementary Table 2. Baseline variables stratified according to PCFS-score**

Details of the patient population at baseline and medical treatment for COVID -19 stratified according to a low and high functional status based on the Post-COVID-19 Functional Status (PCFS)-score. Data are presented a number (percentages) of mean ± standard deviation.

Data are presented a number (percentages) of mean ± standard deviation.

Abbreviations: BB, beta blocker BMI: body mass index, CABG: coronary artery bypass graft, CCB: calcium channel blocker, COPD: chronic obstructive pulmonary disease, CVA: cerebrovascular accident, CVD: cardiovascular disease, HIV: human immunodeficiency virus, HT; hypertension MTx, methotrexate, OSAS: obstructive sleep apnea syndrome, PCI: percutaneous coronary intervention, PVD: Peripheral vascular disease, TIA: transient ischemic attack

| **Characteristic (N=81)** | **Overall** | **No ICU Admission (N= 47)** | **ICU Admission (N= 34)** | **P-value** |
| --- | --- | --- | --- | --- |
| **Laboratory values Admission** |  |  |  |  |
| Lymphocytes number (1·0 – 3·50 10^9/L) | 0·92±0·50 | 0·91±0·54 | 0·92±0·42 | 0·944 |
| Lymphopenia (<1 10^9/L) | 51 (62%) | 31 (66%) | 20 (66%) | 0·949 |
| Elevated liver enzyme (ASAT/ALAT >2UL) | 13(15%) | 6 (13%) | 7 (21%) | 0·356 |
| LDH (<248U/L) | 369  (279-474 | 341  (272-05) | 453  (300-604) | **0·009** |
| Elevated LDH (≥248 U/L) | 64 (78%) | 37(79%) | 27 (79%) | 0·840 |
| Ferritin (10-150 ug/L) | 723 (375-1371 | 723  (276-1266) | 955 (398-1540) | 0·481 |
| Hyperferritineamie (≥150 ug/L) | 42 (52%) | 19 (40%) | 20 (59%) | 0·607 |
| CRP (<5·0 ng/L) | 133±98 | 108±83 | 169±106 | **0·008** |
| Elevated CRP (≥5 ng/L) | 78 (95%) | 45 (96%) | 33 (97%) | 0·230 |
| Maximum Troponin T (<14 ng/L) | 18·3±17·7 | 16±18 | 21±16 | 0·153 |
| Troponin T elevated (≥14 ng/L) | 35 (43%) | 16 (34%) | 19 (56%) | **0·027** |
| Maximum NT-proBNP (<248 ng/L) | 165 (56-612 | 165(65-617) | 175(43-629) | 0·481 |
| NT-ProBNP elevated (>248 ng/L) | 33 (41%) | 18 (38%) | 15 (44%) | 0·421 |
|  |  |  |  |  |
| **Laboratory values Outpatient clinic** |  |  |  |  |
| Lymphocytes number | 2·0±0·8 | 1·77±0·73 | 2·30±0·83 | **0·006** |
| Lymphopenia | 5 (6%) | 5 (12%) | 0 (0%) | 0·057 |
| Elevated liver enzyme | 2 (2%) | 1 (2%) | 1 (3%) | 0·808 |
| LDH | 183±31 | 188±33 | 176±28 | 0·098 |
| Elevated LDH | 2 (2%) | 1 (2%) | 1 (3%) | 0·808 |
| Ferrtin | 191 (79-366) | 158 (198-310) | 206 (47-417) | 0·836 |
| Hyperferritineamie | 41 (49%) | 23 (54%) | 18 (60%) | 0·581 |
| CRP | 2·3±2·9 | 1·8±1·9 | 2·9±4·0 | 0·134 |
| Elevated CRP | 6 (7%) | 2 (5%) | 3 (125) | 0·350 |
| Troponin T | 11 ± 11 | 12·0±13·1 | 11·1±10·4 | 0·755 |
| Troponin T elevated | 15 (19%) | 9 (19%) | 6 (18%) | 0·864 |
| NT-ProBNP | 71 (27-165) | 78 (24-181) | 67 (31-157) | 0·884 |
| NT-ProBNP elevated | 12 (15%) | 8 (17%) | 4 (12%) | 0·493 |
| Total cholesterol (<5·0 mmol/L) | 5·4±1·2 | 5·2±1·1 | 5·5±1·2 | 0·431 |
| LDL-Cholesterol (<3·0 mmol/L) | 3·1±1·1 | 3·2±1·0 | 3·1±1·1 | 0·759 |
| Triglycerides (<2·0 mmol/L) | 2·0±0·9 | 1·7±0·8 | 2·3±1·0 | **0·021** |

**Supplementary Table 3. Laboratory values during admission and at outpatient follow-up for the overall population and stratified according to ICU admission·**

Additional laboratory values of the study population, both during admission and at the outpatient clinic, providing insight in cardiac damage, cardiovascular risk profile and inflammatory response. Data are depicted for the overall population and stratified according to admission to the ICU or general ward. Data are presented a number (percentages) of mean ± standard deviation or median (interquartile range) for non-normally distributed values.

Abbreviations: ALAT: alanine aminotransferase, ASAT: aspartate aminotransferase, CRP: C-reactive protein, LDH: lactate dehydrogenase· NT-proBNP: N-Terminal Pro–B-Type Natriuretic Peptide UL: upper limits of normal.

| **Characteristic (N=81)** | **PCFS <3 (N=44)** | **PCFS ≥3 (N=37)** | **P-value** |
| --- | --- | --- | --- |
| **Laboratory values Admission** |  |  |  |
| Lymphocytes number (1·0 – 3·50 10^9/L) | 0·87±0·38 | 0·97±0·61 | 0·416 |
| Lymphopenia (<1 10^9/L) | 27 (64%) | 24 (69%) | 0·692 |
| Elevated liver enzyme (ASAT/ALAT >2UL) | 7 (16%) | 6 (16%) | 0·919 |
| LDH (<248U/L) | 358 (284-418) | 418(262-550) | 0·33 |
| Elevated LDH (≥248 U/L) | 37 (84%) | 27 (73%) | 0·187 |
| Ferritin (10-150 ug/L) | 1150(559-1351) | 554 (240-1411 | 0·124 |
| Hyperferritineamie (≥150 ug/L) | 20 (100%) | 19 (86%) | 0·087 |
| CRP (<5·0 ng/L) | 142±106 | 123±86 | 0·370 |
| Elevated CRP (≥5 ng/L) | 42 (95%) | 37 (100%) | 0·914 |
| Maximum Troponin T (<14 ng/L) | 17±17 | 19±19 | 0·523 |
| Troponin T elevated (≥14 ng/L) | 16 (36%) | 19 (51%) | 0·122 |
| Maximum NT-proBNP (<248 ng/L) | 122 (44-603 | 193 (92-638) | 0·485 |
| NT-ProBNP elevated (>248 ng/L) | 17 (39%) | 16 (43%) | 0·508 |
|  |  |  |  |
| **Laboratory values Outpatient clinic** |  |  |  |
| Lymphocytes number | 1·93±0·78 | 2·05±0·85 | 0·556 |
| Lymphopenia | 4 (10%) | 1 (3%) | 0·254 |
| Elevated liver enzyme | 1 (2%) | 1 (3%) | 0·905 |
| LDH | 181±32 | 184±30 | 0·706 |
| Elevated LDH | 1 (2%) | 1(3%) | 0·905 |
| Ferritin· | 198 (88-381) | 158 (60-324) | 0·247 |
| Hyperferritineamie | 24 (60%) | 17 (52%) | 0·467 |
| CRP | 2·0±3·0 | 2·5±3·0 | 0·504 |
| Elevated CRP | 2 (5%) | 3 (12%) | 0·350 |
| Troponin T | 10·3±8·6 | 13·1±14·9 | 0·361 |
| Troponin T elevated | 7 (16%) | 8 (22%) | 0·510 |
| NT-ProBNP | 67 (24-123) | 92 (32-184) | 0·397 |
| NT-ProBNP elevated | 6 (14%) | 6 (16%) | 0·788 |
| Total cholesterol (<5·0 mmol/L) | 5·7±1·0 | 5·1±1·3 | 0·072 |
| LDL-Cholesterol (<3·0 mmol/L) | 3·4±1·0 | 2·9±1·1 | 0·082 |
| Triglycerides (<2·0 mmol/L) | 2·0±1·0 | 2·1±1·0 | 0·589 |

**Supplementary Table 4. Laboratory values during admission and at outpatient follow-up stratified according to PCFS-score.**

Additional laboratory values of the study population, both during admission and at the outpatient clinic, providing insight in cardiac damage, cardiovascular risk profile and inflammatory response. Data are stratified according to a low and high functional status based on the Post-COVID-19 Functional Status (PCFS)-score. Data are presented a number (percentages) of mean ± standard deviation or median (interquartile range) for non-normally distributed values.

Abbreviations: ALAT: alanine aminotransferase, ASAT: aspartate aminotransferase, CRP: C-reactive protein, LDH: lactate dehydrogenase· NT-proBNP: N-Terminal Pro–B-Type Natriuretic Peptide UL: upper limits of normal.

**Supplementary Table 5. Convergent construct validity of the PCFS score·**

The convergent construct validity of the PCFS score was investigated in a subsample of 57 of the 81 patients with the 5 dimensions of the EQ-5D-5L and the patient’s self-rated health score using Spearman-rho correlation coefficient:

| **EQ-5D-5L dimension** | **Spearman-rho correlation coefficient with PCFS-score** |
| --- | --- |
| Self-rated health score | -0·55 |
| Mobility | 0·41 |
| Self-care | 0·50 |
| Usual activities | 0·47 |
| Pain/discomfort | 0·37 |
| Anxiety/depression | 0·31 |
