# Supplementary material for: Short-term outpatient follow-up of COVID-19 patients: A multidisciplinary approach
Source: eClinicalMedicine. 2021 Jan 28;32:100731. doi: 10.1016/j.eclinm.2021.100731 (PMC7843037; doi:10.1016/j.eclinm.2021.100731)
Supplement: Supplementary file 2 [file mmc2.docx]

***Psychological questionnaires***

**Anxiety** is assessed with the generalized anxiety disorder questionnaire (GAD-7). The 7-item GAD is a self-report questionnaire and used as a screening tool and severity measure for anxiety according to generalized anxiety disorder of the DSM-IV.^1,2^ The items are scored on a scale from 0 meaning ‘Not at all’ to 3 indicating ‘Almost each day’. A high total score refers to a possible anxiety disorder with a maximum score of 21. A cut-off score for moderate-severe anxiety > 10 is used.^3^ The internal consistency within the current sample is good (Cronbach's α = 0.90).

**Depression** is assessed with the patient health questionnaire (PHQ-9). ^4^ The 9-item PHQ is a self-report questionnaire that screens depressive symptomatology in accordance with DSM-IV criteria. Items can be scored from 0 ‘Not at all’ to 3 ‘Almost each day’ with a maximum of 27. A high score suggests more depressive symptoms. A cut-off score for moderate-severe depression > 10 is used. ^5^ The internal consistency of the PHQ-total score within the current sample is good (Cronbach's α = 0.86).

**Post-Traumatic Stress Symptoms** are assessed with the PTSD Checklist for DSM-5 (PCL-5). The PCL is a 20-item self-report measure to screen for PTSD symptoms according to the DSM-5 criteria.^6^ Items are scored on a Likert scale ranging from 0 to 4, where higher scores indicate more pronounced PTSD symptoms. The sum of scores can range from 0 to 80. A cut-off score of 38 and higher is suggested to indicate PTSD. The internal consistency of the PCL total score within the current sample is good (Cronbach's α = 0.95).

**Cognitive function** is assessed with the Cognitive Failures Questionnaire (CFQ) and an adapted version of the Informant Questionnaire on Cognitive Functioning in the Elderly (IQ-CODE-N) ^7,8^

The *CFQ* is a 25-item self-report measure and assess frequency of failures in perception/attention (e.g., Do you fail to notice signposts on the road?‘), memory (e.g., Do you forget appointments?‘), and action (e.g., Do you bump into people?‘). Items are scored on a 5-point Likert scale ranging from 0 to 4 from ‘never’’ to ‘very often’.^7^ A high total score refers to a higher frequency of cognitive failures, with a maximum of 100. The cut-off score of > 31 was used for cognitive failure. The internal consistency of the total CFQ score within the current sample is good (Cronbach's α = 0.95).

The *IQ-CODE-N* is a 16-item questionnaire completed by the care-giver.^8^ It asks for changes in patient’s everyday cognitive function (including finances, communication, memory, household appliances) following Covid-19. Items are scored on a 5-points Likert scale ranging from much better (1), not much change (3) to much worse (5). A mean total score > 3 indicates a worsening of cognitive function since the covid-19 infection. A mean total score < 3 indicates better cognitive function.

References

1. Donker T, van Straten A, Marks I, Cuijpers P. Quick and easy self-rating of Generalized Anxiety Disorder: validity of the Dutch web-based GAD-7, GAD-2 and GAD-SI. *Psychiatry Res* 2011; **188**(1): 58-64.

2. Spitzer RL, Kroenke K, Williams JB, Löwe B. A brief measure for assessing generalized anxiety disorder: the GAD-7. *Arch Intern Med* 2006; **166**(10): 1092-7.

3. Plummer F, Manea L, Trepel D, McMillan D. Screening for anxiety disorders with the GAD-7 and GAD-2: a systematic review and diagnostic metaanalysis. *Gen Hosp Psychiatry* 2016; **39**: 24-31.

4. Kroenke K, Spitzer RL, Williams JB. The PHQ-9: validity of a brief depression severity measure. *J Gen Intern Med* 2001; **16**(9): 606-13.

5. Levis B, Benedetti A, Thombs BD. Accuracy of Patient Health Questionnaire-9 (PHQ-9) for screening to detect major depression: individual participant data meta-analysis. *Bmj* 2019; **365**: l1476.

6. Blevins CA, Weathers FW, Davis MT, Witte TK, Domino JL. The Posttraumatic Stress Disorder Checklist for DSM-5 (PCL-5): Development and Initial Psychometric Evaluation. *J Trauma Stress* 2015; **28**(6): 489-98.

7. Broadbent DE, Cooper PF, FitzGerald P, Parkes KR. The Cognitive Failures Questionnaire (CFQ) and its correlates. *Br J Clin Psychol* 1982; **21**(1): 1-16.

8. Jorm AF, Jacomb PA. The Informant Questionnaire on Cognitive Decline in the Elderly (IQCODE): socio-demographic correlates, reliability, validity and some norms. *Psychol Med* 1989; **19**(4): 1015-22.
